# Performance of multiparametric prostate magnetic resonance imaging validated by targeted and systematic transperineal biopsies

**DOI:** 10.1002/bco2.184

**Published:** 2022-08-19

**Authors:** Richard A. Hsi, Tru‐Khang Dinh, Matthew Greer, Carleen Bensen, Marc A. Mitchell, Amy Y. Li, Andrew Stamm, Manfred Henne

**Affiliations:** ^1^ Seattle Cancer Care Alliance Peninsula Poulsbo Washington USA; ^2^ University of Washington Seattle Washington USA; ^3^ Olympic Medical Center Port Angeles Washington USA; ^4^ The Doctors Clinic Silverdale Washington USA; ^5^ Rayus Imaging Poulsbo Washington USA

**Keywords:** MR‐guided biopsy, multiparametric prostate MRI, PI‐RADS score, prostate cancer, transperineal biopsy

## Abstract

**Objective:**

To measure the performance of multiparametric (mp) magnetic resonance imaging (MRI) to identify intraprostatic tumour deposits using a systematic and targeted MR‐guided transperineal prostate biopsy technique.

**Materials and Methods:**

Patients underwent a combined systematic and targeted MR‐guided transperineal biopsy procedure in the dorsal lithotomy position under general anaesthesia. Systematic biopsies were spaced 10 mm or less apart and additional biopsies targeted any Prostate Imaging–Reporting and Data System (PI‐RADS) 3, 4 or 5 lesions identified on mpMRI. Cancer detection rates were calculated on a per patient and per lesion basis.

**Results:**

A total of 125 patients underwent the biopsy procedure. The positive predictive value (PPV) of mpMRI per patient was 59% for any cancer and 49% for Gleason score (GS) ≥ 7 cancer. The negative predictive value (NPV) of mpMRI per patient was 67% for any cancer and 88% for GS ≥ 7 cancer. On a per lesion basis, the PPV of PI‐RADS 3 lesions for any and GS ≥ 7 cancer was 24% and 10%. For PI‐RADS 4 lesions it was 42% and 32%. For PI‐RADS 5 lesions, it was 76% and 70%. MpMRI failed to identify GS ≥ 7 cancer found on systematic biopsy in 22% of patients.

**Conclusion:**

Based on a combination of systematic and targeted transperineal prostate biopsies, mpMRI showed a high NPV and low PPV for GS ≥ 7 cancer on a per patient basis. The PPV of mpMRI on a per lesion basis increased with increasing PI‐RADS score. However, there were a significant number of both false positive as well as false negative (mpMRI invisible) areas within the prostate that contained GS ≥ 7 cancer. Therefore, pathologic confirmation using both targeted and systematic mapping biopsy is necessary to accurately identify all intraprostatic tumour deposits.

## 1 INTRODUCTION

The introduction of multiparametric magnetic resonance imaging (mpMRI) scans of the prostate has dramatically changed the diagnostic pathway for prostate cancer over the last decade. Current NCCN (National Comprehensive Cancer Network) guidelines include consideration of mpMRI and targeted MR fusion prostate biopsy as acceptable options for the prostate cancer screening process (NCCN Early Detection Guidelines). In response to the widespread adoption of mpMRI into clinical practice and excessive variation in interpretation and reporting, a standardized reporting system was introduced in 2012 known as PI‐RADS (Prostate Imaging–Reporting and Data System) version 1 and updated to PI‐RADS version 2 in 2015.^1^, ^2^ Studies utilizing this system showed the use of mpMRI can, in general, lead to a reduction in overdiagnosis of clinically indolent prostate cancer and improved detection of clinically significant cancers.^3^, ^4^

Most of the reports on the performance of mpMRI, however, have focused on a per patient rather than per lesion basis in order to measure its diagnostic reliability. Extensive analyses of the sensitivity, specificity, positive and negative predictive values (PPV and NPV) to establish the presence or absence of prostate cancer in a given patient have been reported.^5^ If the assumption is that patients diagnosed with prostate cancer who require treatment (i.e., those with clinically significant disease) will undergo whole gland therapy, the delineation of all tumours within a prostate is not critical. However, radical therapy such as surgical removal or radiation therapy can result in significant urinary, bowel and sexual dysfunction. As a result, there has been increased interest in focal prostate therapy in an effort to control tumour progression while avoiding or minimizing the toxicities associated with whole gland therapy. Over the past 15 years, reports have been published describing the use of focal therapy either as primary treatment, as an intraprostatic boost for whole gland therapy and as salvage treatment for locally recurrent prostate cancer.^6^, ^7^, ^8^ Many of these studies utilized mpMRI to delineate treatment targets within the prostate. As current and future clinical trials continue to incorporate mpMRI into their treatment strategies, it will be important to take into consideration the abilities and limitations of these scans in identification of tumour within the prostate.

The purpose of this study was to evaluate the performance of prostate mpMRI on both a per patient and per lesion basis validated by targeted and systematic transperineal prostate biopsies.

## 2 MATERIALS AND METHODS

### 2.1 Patient population

In this study, the data from patients in a community practice setting undergoing mpMRI followed by MR fusion transperineal prostate biopsy from 2015 through 2021 were collected. Subjects undergoing both initial and repeat prostate biopsy after initial transrectal ultrasound guided (TRUS) biopsy were included. IRB approval was obtained to retrospectively analyse the records of each patient to obtain prebiopsy characteristics, mpMRI results, biopsy procedure details and pathology reports of the biopsy specimens. Patients were excluded if they could not receive gadolinium contrast or had undergone prior MRI fusion biopsy or saturation biopsy. Patients with hip replacement were also excluded due to extensive susceptibility artefact and image distortion on MRI, which could preclude reliable identification and delineation of PI‐RADS lesions. In total, a final cohort of 125 patients met the study criteria.

### 2.2 MRI protocol and interpretation

All mpMRIs were obtained using a 3 Tesla (3 T) scanner (MAGNETOM Verio, Siemens Healthineers, Erlangen Germany) with a pelvic phased‐array coil. No endorectal coil was used. The imaging protocol included T2‐weighted imaging (T2WI), diffusion‐weighted imaging (DWI) and dynamic contrast‐enhanced imaging (DCE).

All studies were interpreted by a single radiologist (MH) at the diagnostic radiology facility housing the MRI scanner. This physician had 25 years of clinical radiology experience prior to initiation of this procedure. MRI scans were read as part of routine clinical care using PI‐RADS v2. No standardized training or formal performance feedback was given prior to or during the study period.

### 2.3 Transperineal MRI‐fusion prostate biopsy protocol

**FIGURE 1:**
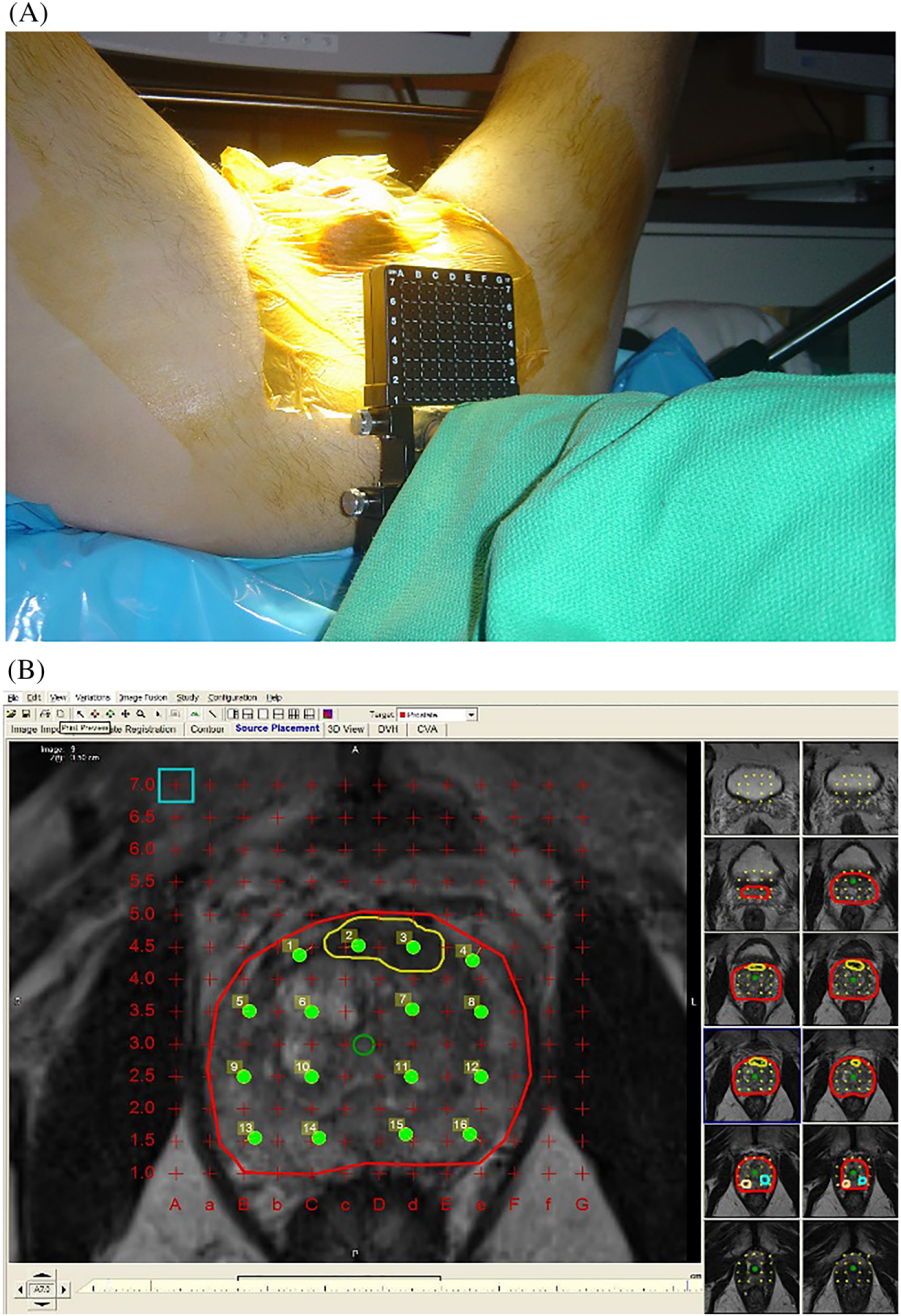
(A) Transperineal biopsy set‐up. (B) Transperineal biopsy plan

All biopsies were performed by a single physician (RAH). This physician is a radiation oncologist who is a brachytherapy specialist with 18 years of clinical experience prior to initiation of this procedure. All PI‐RADS 3, 4 and 5 lesions were targeted based on results of the imaging from the above mpMRI protocol. Targets were confirmed by demarcation by the radiologist on captured key images available in the radiology facility PACS system or by personal review by RAH with the radiologist. Biopsy planning was performed by importing T2‐weighted images into a commercially available transperineal biopsy planning system (Variseed/Varipath, Varian Medical Systems, Palo Alto, CA, USA). Images were then reoriented from supine (position mpMRI images were obtained) to dorsal lithotomy (position in which the transperineal biopsies were performed). The prostate was divided into an apical and base half and systematic biopsies were then planned 10 mm or less apart (from each other, the edge of the prostate or the urethra) for each half. Additional biopsies were also planned for each PI‐RADS 3, 4 or 5 lesion. When no lesions were identified on mpMRI, only systematic biopsies were performed. Rigid fusion was used and confirmed using anatomic landmarks referenced to a transperineal template grid (see Figure 1). All biopsies were carried out in the dorsal lithotomy position under general anaesthesia. A digital ultrasound with transrectal probe (BK Medical, Peadbody, MA, USA), ultrasound stepper stabilizer and transperineal template grid (Civco, Coralville, IA, USA) were used for each biopsy procedure. Tissues cores were sent for histopathologic review and clinically significant prostate cancer was defined as Gleason score ≥7 adenocarcinoma.

All patients received oral antibiotic prophylaxis for 3 days (the day prior, of and after the biopsy procedure) and a bowel prep consisting of clear liquids the day prior and Fleet® enemas the night before and morning of the procedure. All patients had a urinary catheter placed during the procedure to identify the urethra.

### 2.4 Radiographic pathologic correlation methodology

Biopsy specimens were read by genitourinary pathologists at either of two large multispecialty laboratories. The identified PI‐RADS lesions were called positive if any of the biopsies from within the outlined lesions contained carcinoma. The PI‐RADS lesions were also called positive if any biopsy outside but within 5 mm of the outlined lesion contained carcinoma. Due to core sampling overlap between the apical and basal half of the gland, if cores were positive from the identical grid position in both the apical and basal half of the gland, they were not counted as separate lesions.

### 2.5 Statistical analysis

Descriptive statistics were used to present patient characteristics and cancer detection rates. We reviewed the cancer detections rates on a per patient and individual lesion basis. We determined the PPV and NPV of the PI‐RADS v2 system in this series. The 95% confidence intervals (95% CI) were calculated for all proportional outcomes.

## 3 RESULTS

### 3.1 Patient characteristics—See Table 1

**TABLE 1:** Patient characteristics

No. of Patients	125
No prior Bx	60
Negative prior Bx	65
Median age (range, IQR) years	66 (45–87, 62–72)
Median PSA (range, IQR) ng/ml	7.4 (0.5–46.1, 5.9–10.0)
Median volume (range, IQR) cc	53 (21–170, 40–73)
No. of targeted lesions	161
PI‐RADS 3	41
PI‐RADS 4	74
PI‐RADS 5	46
Median no. of Bxs (range, IQR)	25 (16–44, 21–31)

Abbreviation: IQR, interquartile range; PI‐RADS, Prostate Imaging–Reporting and Data System.

The median patient age was 66 years (range 45–87 years, interquartile range [IQR] 62–72 years) with a median PSA of 7.4 ng/ml (range 0.5–46.1 ng/ml, IQR 5.9–10.0 ng/ml). The median number of biopsies obtained per patient was 25 (range 16–44, IQR 21–31). The median prostate volume was 53 cc (range 21–170 cc, IQR 40–73 cc). Among 125 patients undergoing biopsy procedure, 60 were biopsy naïve (Group 1) and 65 had undergone prior negative transrectal biopsy (Group 2).

### 3.2 Per patient analysis

A total of 101 patients had a mpMRI with at least one identified PI‐RADS 3, 4 or 5 lesion. A total of 24 patients had no PI‐RADS 3, 4 or 5 identified lesions on mpMRI.

The NPV per patient in this series was 67% (16/24, 95% CI 0.45–0.84) for any cancer and 88% (21/24, 95% CI 0.68–0.97) for GS ≥ 7 cancer. The PPV per patient in this series was 59% (60/101, 95% CI 0.49–0.69) for any cancer and 49% (49/101, 95% CI 0.38–0.59) for GS ≥ 7 cancer.

Prostate cancer was identified by systematic biopsy outside of any PI‐RADS 3, 4 or 5 lesion in 40% (50/125, 95% CI 0.31–0.49) of patients (mpMRI invisible lesions). In addition, mpMRI invisible GS ≥ 7 cancer was found in 22% (28/125, 95% CI0.15–0.31) of patients. MpMRI invisible GS ≥ 4 + 3 = 7 cancer was found in 10% (13/125, 95% CI 0.06–0.17), and GS ≥ 8 cancer was found in 5% (6/125, 95% CI 0.02–0.10) of patients. MpMRI invisible GS ≥ 7 cancer with no other GS ≥ 7 mpMRI identified cancer elsewhere within the prostate was found in just 6% (7/125, 95% CI 0.02–0.11) of patients.

### 3.3 Per lesion analysis

A total of 41 PI‐RADS 3, 74 PI‐RADS 4 and 46 PI‐RADS 5 lesions were identified on mpMRI (see Table 2, which includes 95% CI for each proportion). The overall PPV of all PI‐RADS 3, 4 and 5 lesions was 47% (76/161) for any cancer and 37% (60/161) for GS ≥ 7 cancer. For PI‐RADS 3 lesions, the PPV for any and GS ≥ 7 cancer was 24% (10/41) and 10% (4/41). For PI‐RADS 4 lesions it was 42% (31/74) and 32% (24/74). For PI‐RADS 5 lesions it was 76% (35/46) and 70% (32/46).

**TABLE 2:** Positive predictive value of PI‐RADS lesions

Overall					
PI‐RADS score	No. of lesions	Positive—Any GS (%)	95% CI	Positive GS ≥ 7 (%)	95% CI
3, 4 or 5	161	47	0.39–0.55	37	0.30–0.45
3	41	24	0.12–0.40	10	0.03–0.23
4	74	42	0.31–0.54	32	0.22–0.44
5	46	76	0.61–0.87	70	0.54–0.82

Group 1					
PI‐RADS score	No. of lesions	Positive—Any GS (%)	95% CI	Positive GS ≥ 7 (%)	95% CI
3, 4 or 5	78	62	0.50–0.72	44	0.32–0.55
3	21	38	0.18–0.62	14	0.03–0.36
4	38	61	0.43–0.76	42	0.26–0.59
5	19	89	0.67–0.99	79	0.54–0.94

Group 2					
PI‐RADS score	No. of lesions	Positive—Any (%)	95% CI	Positive GS ≥ 7 (%)	95% CI
3, 4 or 5	83	35	0.25–0.46	31	0.22–0.42
3	20	10	0.01–0.31	5	0.00–0.25
4	36	25	0.12–0.42	22	0.10–0.39
5	27	67	0.46–0.83	63	0.42–0.81

*Note*: Group 1 = biopsy naïve; Group 2 = prior negative biopsy.

Abbreviations: CI, confidence interval; GS, Gleason score; PI‐RADS, Prostate Imaging–Reporting and Data System.

In the biopsy naïve patients (Group 1), a total of 21 PI‐RADS 3, 38 PI‐RADS 4, and 19 PI‐RADS 5 lesions were identified. The overall PPV of all PI‐RADS 3, 4 and 5 lesions in Group 1 was 62% (48/78) for any cancer and 44% (34/78) for GS ≥ 7 cancer. The PPV for any and GS ≥ 7 cancer for PI‐RADS 3 lesions was 38% (8/21) and 14% (3/21). The PPV for any and GS ≥ 7 cancer for PI‐RADS 4 lesions was 61% (23/38) and 42% (16/38). The PPV for any and GS ≥ 7 cancer for PI‐RADS 5 lesions was 89% (17/19) and 79% (15/19).

In the patients with a prior negative transrectal biopsy (Group 2), a total of 20 PI‐RADS 3, 36 PI‐RADS 4, and 27 PI‐RADS 5 lesions were identified. The overall PPV of all PI‐RADS 3, 4 and 5 lesions in Group 2 was 35% (29/83) for any cancer and 31% (26/83) for GS ≥ 7 cancer. The PPV for any and GS ≥ 7 cancer for PI‐RADS 3 lesions was 10% (2/20) and 5% (1/20). The PPV for any and GS ≥ 7 cancer for PI‐RADS 4 lesions was 25% (9/36) and 22% (8/36). The PPV for any and GS ≥ 7 cancer for PI‐RADS 5 lesions was 67% (18/27) and 63% (17/27).

## 4 DISCUSSION

The utilization of mpMRI has expanded from diagnosis and screening to surgical and radiation therapy treatment planning and new treatment approaches such as focal and focal boost therapy. Despite widespread adoption of mpMRI, its statistical performance on a per patient and more so on a per lesion basis, remains somewhat variable.

This study, in summary, evaluated the performance of mpMRI on a per patient and per lesion basis in patients undergoing systematic and targeted transperineal prostate biopsy using the PI‐RADS v2 system.

### 4.1 Per patient analysis

We evaluated the positive and NPV of mpMRI in this study on a per patient basis to study its performance as a diagnostic tool. Large multi‐institutional trials such as the PROMIS trial^9^ have demonstrated that mpMRI can increase the number of clinically significant (Gleason score ≥4 + 3 = 7 or maximum cancer core length ≥ 6 mm) prostate cancers diagnosed and potentially reduce the number of patients diagnosed with clinically insignificant disease compared to TRUS prostate biopsy alone and suggest mpMRI could be used as a triage test for patients with an elevated PSA. Current NCCN guidelines now include mpMRI as an option for prebiopsy testing in patients with an elevated PSA.

The accuracy of this modality in terms of both NPV and PPV is the subject of multiple retrospective studies. A large meta‐analysis by Sathianathan et al. calculated a pooled NPV of 90.8% for biopsy naïve patients and 92.7% for patients with prior negative biopsy. There was no statistical difference between groups.^10^ These numbers compare favourably with our findings of a NPV of 88% for the combined Groups 1 and 2 using the same definition of negative mpMRI (PI‐RADS score <3) and clinically significant prostate cancer (GS ≥ 7). Thus, the NPV of mpMRI is high, although approximately 1 in 10 cases of clinically significant prostate cancer would be missed if biopsy was omitted. Another large study by Westphalen et al. examined the PPV mpMRI from combined data submitted from 26 institutions involving 3449 patients who were either biopsy naïve, had a prior negative biopsy or had prior biopsy proven prostate cancer.^11^ The PPV of mpMRI on a per patient basis was 60% for any cancer and 41% for GS ≥ 7 cancer. Our results compare favourably with a PPV of 59% for any cancer of 49% for GS ≥ 7 cancer. Of note, our series does not include patients with prior biopsy proven prostate cancer. Similar series to ours that include only biopsy naïve and prior negative biopsy patients, however, report similar PPV outcomes.^12^, ^13^, ^14^

Thus, the relatively high NPV and low PPV of mpMRI found in the current study seems to be consistent with other published series and confirms the conclusion that mpMRI is a relatively good screening tool to avoid biopsy, but improvements must still be made to more reliably and consistently predict the presence of clinically significant prostate cancer.

### 4.2 Per lesion analysis

We evaluated the PPV of mpMRI in this study on a per lesion basis to study its performance in correct identification of intraprostatic tumour deposits. The PPV increased with increasing PI‐RADS score both for any cancer (PI‐RADS 3 = 24%, PI‐RADS 4 = 42%, and PI‐RADS 5 = 76%) and GS ≥ 7 cancer (PI‐RADS 3 = 10%, PI‐RADS 4 = 32%, and PI‐RADS 5 = 70%). A review of the literature (Table 3) shows that the patient groups, PI‐RADS version used, biopsy route and reference standards vary widely across studies. Additionally, the expertise of each institution as well as individuals both reading the MRI scans and performing the biopsy procedures, the MRI fusion technology (cognitive, fusion software, in bore), and MRI hardware variability could play into these results. However, despite these variations, there is relative consistency in PPV for both any prostate cancer as well as clinically significant prostate cancer with increasing PI‐RADS score.

**TABLE 3:** Studies reporting positive predictive value of PI‐RADS system

Author	No. patients	Patient groups	PI‐RADS version	Biopsy technique	Reference standard	% Positive PI‐RADS 3		% Positive PI‐RADS 4		% Positive PI‐RADS 5	
						Any GS	GS ≥ 7	Any GS	GS ≥ 7	Any GS	GS ≥ 7
Hsi (current)	125	1,2	V2	TP	TP 25 core (median)	24	10	42	32	76	70
Ting^26^	80	1,2,3	V1	TP	TP 24 core (median)	38	21	69	60	91	82
Marra^12^	1014	1,2	V2	TP	TP 12 core	20	20	51	41	78	70
Hansen^27^	487	2	V1	TP	TP 24 core (median)	44	20	58	32	83	70
Mannaerts^13^	255	1,2	V2	TRUS	TRUS 12 core	25	17	53	46	80	70
Mehralivand^14^	339	1,2	V2	TRUS	TRUS 12 core	25	12	39	22	87	72
Venderink^28^	1057	1,2,3	V1 and V2	TRUS	None	35	17	60	34	91	67
Sonn^29^	409	1,2,3	V1 and V2	TRUS	TRUS 12.4 core (median)	24	12	55	38	75	63
Wysock^30^	125	1,2,3	V1	TRUS	TRUS 12 core	23	8	42	32	86	81

*Note*: Group 1 = biopsy naïve; Group 2 = prior negative biopsy; Group 3 = prior prostate cancer diagnosis.

Abbreviations: GS, Gleason score; PI‐RADS, Prostate Imaging–Reporting and Data System; TP, transperineal; TRUS, transrectal ultrasound‐guided.

Of note, in our series, there did appear to be a lower PPV for each PI‐RADS category for patients with a prior negative prostate biopsy (Group 2) versus patients undergoing their first biopsy. For GS ≥ 7 disease, the PPV for Group 2 was 5%, 22% and 63% for PI‐RADS 3, 4 and 5 lesions, respectively. For Group 1 it was 14%, 42% and 79%, respectively. This difference seems logical as Group 2 would be statistically biased towards a negative result. Prior studies have also demonstrated a higher PPV in first biopsy patients compared to those with a prior negative TRUS biopsy.^15^, ^16^

Although the PI‐RADS system was not designed to guide prostate cancer treatment, mpMRI has been utilized to guide intraprostatic treatment both in the form of primary focal and focal boost therapy.^6^, ^7^ Yet, based on our results, it is clear that only approximately 40% of PI‐RADS ≥ 3 lesions contain clinically significant cancer and only 50% contain any cancer at all. These results would suggest that treatment based on mpMRI information alone would result in a large number of patients receiving treatment or dose escalated treatment to benign or clinically insignificant disease. In addition, other studies have shown that the delineation of MR targets based on MRI can be quite variable among individual practitioners and pathologic data suggest that MRI may underestimate true tumour volume as well.^17^, ^18^ Such limitations would suggest that all patients should have biopsy confirmation of mpMRI identified lesions before treatment and that more formal target contouring guidelines including the addition of extra margin around the lesion need to be developed.

### 4.3 mpMRI invisible prostate cancer

The effect of unidentified (mpMRI invisible) tumour has differing relevance depending upon whether the imaging is utilized for diagnosis or guidance for treatment decisions. In this current study, 22% of patients were found to have mpMRI invisible GS ≥ 7 disease. Other reports have found similar rates of mpMRI invisible tumour ranging from 10% to 28%.^18^, ^19^, ^20^ However, in our series, only 6% of patients had mpMRI invisible GS ≥ 7 prostate cancer without other same or higher grade tumours identified elsewhere within the same prostate. Thus, from purely diagnostic standpoint, 94% of patients with clinically significant cancer somewhere within the prostate were identified by mpMRI. This high rate of detection would seem acceptable if mpMRI is used as a tool to help screen and perhaps direct prostate biopsy when trying to establish the diagnosis of GS ≥ 7 prostate cancer.

However, the presence of mpMRI invisible prostate cancer may have more significance when using mpMRI to guide focal therapy both as primary as well as intraprostatic boost treatment. The rationale for using focal therapy in an attempt to reduce toxicity of whole gland prostate treatment is based on the hypothesis that an index lesion (the largest tumour focus within the prostate) drives the natural history of an individual's prostate cancer and that anatomically distinct metastatic foci within a patient may share a monoclonal origin from a single progenitor found in that index lesion.^21^ However, this hypothesis is far from proven and other data would suggest that non index lesions can be the origin of both local invasion with extracapsular spread and as well as metastatic disease.^22^, ^23^ In a study by Le et al, 20% of patients were found to have non index tumours that were GS ≥ 7 and 6% of patients had GS ≥ 4 + 3 = 7 non index tumours, the majority of which were not seen on MRI.^20^ These findings, combined with the current study results showing 22%, 10% and 5% of patients with GS ≥ 7, GS ≥ 4 + 3 = 7, and GS ≥ 8 unidentified tumours, suggest that a small, but significant number of tumours with the potential for local invasion and metastasis would be missed if intraprostatic disease was assessed by MRI without systematic biopsy.

Despite the above findings, there is a growing number of reports of primary focal and focal boost treatment with a wide variety of treatment modalities, the majority of which utilize MRI to guide treatment.^6^, ^24^ Although results are promising, follow up is short and the lack of rigour around identification of all clinically significant cancer may, in the end, make cancer control rates difficult to interpret. Perhaps the most interesting report is an MRI guided focal boost study by Kerkmeijer et al. (FLAME study), which compared standard external beam radiation therapy to 77 Gy with or without a simultaneous integrated focal boost to 95 Gy in patients with intermediate and high risk prostate cancer. The 5‐year biochemical disease free survival was 7% higher in the focal boost cohort with no difference in prostate specific or overall survival.^7^ Again, follow up is short, but it could be postulated that with histologic confirmation of the boost target, the outcomes might have been even better in favour of the boost arm.

### 4.4 Strengths and limitations

The limitations of this study are first and foremost in its retrospective nature. Obvious referral bias could affect outcomes. This report consisted of patient populations that had either no prior prostate biopsy or prior negative biopsies. Although the PPV and NPV rates are similar to those reported in the literature, the differences between groups are notable. However, many reports as seen in Table 3 also include the same mixed group of patients. This variety of patients, however, also represents the reality of clinical practice. Finally, the definition of clinically significant prostate cancer (GS ≥ 7) is somewhat arbitrary. Volume of the identified lesions was also not specifically taken into account, although the PI‐RADS system does partially account for this issue as the main difference between PI‐RADS 4 and 5 lesions is lesion size >1.5 cm. Also, the present analysis did not take into account the presence of high volume Gleason score 6 disease or patients with Gleason 6 disease with a very high PSA (e.g., >20). These situations may also represent ‘clinically significant’ prostate cancer.

The strengths of this study include the fact that all MRI scans were reviewed by a single radiologist (MH) and all procedures were carried out by a single radiation oncologist (RAH) in a community setting with results comparable to other published series both in terms of radiographic and pathologic correlation. However, each physician did have >18 years of clinical experience prior to initiation of this programme and the radiation oncologist is a specialist in prostate brachytherapy. Therefore, this clinical experience and expertise could also be considered a limitation as it may not be generalizable to other programs. Another strength of this series is that all patients underwent the same MRI protocol and all interpretations used the same PI‐RADS v2 reporting system. In addition, the same biopsy planning software and equipment was used for all biopsy procedures. The reference standard was a systematic transperineal technique, which has been shown to be an effective sampling system, particularly of the anterior prostate when compared to a transrectal approach.^25^ Although no whole mount prostatectomy specimens were available for correlation, it could be argued that using a reference with previously established cancer also introduces bias. Finally, the template guided system used in this series has the potential to be translated into a focal therapy plan. Although the mpMRI images may underestimate tumour volume, if systematic and target biopsies are utilized, a treatment target volume could be generated from the negative cores around the pathologically positive cores thus creating a pathologically confirmed margin around the target.^18^

## 5 CONCLUSION

Based on a combination of systematic and targeted transperineal prostate biopsies, mpMRI showed a high NPV but did miss a small (about 10%) number of patients with GS ≥ 7 prostate cancer. The PPV for GS ≥ 7 prostate cancer was relatively low on a per patient basis. The PPV of mpMRI on a per lesion basis increased with increasing PI‐RADS score and was consistent with other reports in the literature. However, there were a significant number of both false positive as well as false negative (mpMRI invisible) areas within the prostate that contained GS ≥ 7 prostate cancer. Therefore, it would not be advisable to rely solely on mpMRI to target areas within the prostate and pathologic confirmation using both targeted and systematic mapping biopsy prior to focal therapy procedures should be performed.

## CONFLICT OF INTEREST

Richard A. Hsi—Boston Scientific, Provides professional expertise regarding use of SpaceOAR hydrogel, payment made to Hsi.

## AUTHOR CONTRIBUTIONS

Study concept and design: Hsi. Acquisition of data: Henne, Hsi, Bensen, Mitchell, Li, and Stamm. Analysis and interpretation of data: Hsi, Dinh, and Greer. Drafting of manuscript: Hsi. Critical manuscript revision: Dinh and Greer.
